# Initial study on TMPRSS2 p.Val160Met genetic variant in COVID-19 patients

**DOI:** 10.1186/s40246-021-00330-7

**Published:** 2021-05-17

**Authors:** Laksmi Wulandari, Berliana Hamidah, Cennikon Pakpahan, Nevy Shinta Damayanti, Neneng Dewi Kurniati, Christophorus Oetama Adiatmaja, Monica Rizky Wigianita, Soedarsono, Dominicus Husada, Damayanti Tinduh, Cita Rosita Sigit Prakoeswa, Anang Endaryanto, Ni Nyoman Tri Puspaningsih, Yasuko Mori, Maria Inge Lusida, Kazufumi Shimizu, Delvac Oceandy

**Affiliations:** 1 Department of Pulmonology and Respiratory Medicine, Faculty of Medicine, Universitas Airlangga/Dr Soetomo General Academic Hospital, Surabaya, Indonesia; 2 Department of Biomedical Sciences, Faculty of Medicine, Universitas Airlangga, Surabaya, Indonesia; 3 Andrology Program, Faculty of Medicine, Universitas Airlangga, Surabaya, Indonesia; 4 Indrapura KOGABWILHAN II Hospital, Surabaya, Indonesia; 5 Department of Medical Microbiology, Faculty of Medicine, Universitas Airlangga/Clinical Microbiology Unit, Central Laboratory Installation, Dr Soetomo General Academic Hospital, Surabaya, Indonesia; 6 Faculty of Medicine, Universitas Airlangga, Surabaya, Indonesia; 7 Clinical Pathology Program, Faculty of Medicine, Universitas Airlangga, Surabaya, Indonesia; 8 Department of Child Health, Faculty of Medicine, Universitas Airlangga/Dr Soetomo General Academic Hospital, Surabaya, Indonesia; 9 Department of Physical Medicine and Rehabilitation, Faculty of Medicine, Universitas Airlangga/Dr Soetomo General Academic Hospital, Surabaya, Indonesia; 10 Department of Dermatology Venerology, Faculty of Medicine, Universitas Airlangga/Dr. Soetomo General Academic Hospital, Surabaya, Indonesia; 11 Department of Chemistry, Faculty of Science and Technology, Universitas Airlangga, Surabaya, Indonesia; 12 Laboratory of Proteomic, University CoE-Research Center for Bio-Molecule Engineering, Universitas Airlangga, Surabaya, Indonesia; 13 Center for Infectious Diseases, Kobe University Graduate School of Medicine, Kusunoki-cho, Chuo-ku, Kobe, Japan; 14 Institute of Tropical Disease, Universitas Airlangga, Surabaya, Indonesia; 15 Department of Microbiology, Faculty of Medicine, Universitas Airlangga, Surabaya, Indonesia; 16 CRC-ERID, Institute of Tropical Disease, Universitas Airlangga, Surabaya, Indonesia; 17 Division of Cardiovascular Sciences, Faculty of Biology, Medicine and Health, Manchester Academic Health Science Centre, The University of Manchester, Manchester, UK

**Keywords:** COVID-19, TMPRSS2, Polymorphism

## Abstract

**Background:**

Coronavirus disease 2019 (COVID-19) is a global health problem that causes millions of deaths worldwide. The clinical manifestation of COVID-19 widely varies from asymptomatic infection to severe pneumonia and systemic inflammatory disease. It is thought that host genetic variability may affect the host’s response to the virus infection and thus cause severity of the disease. The SARS-CoV-2 virus requires interaction with its receptor complex in the host cells before infection. The transmembrane protease serine 2 (TMPRSS2) has been identified as one of the key molecules involved in SARS-CoV-2 virus receptor binding and cell invasion. Therefore, in this study, we investigated the correlation between a genetic variant within the human *TMPRSS2* gene and COVID-19 severity and viral load.

**Results:**

We genotyped 95 patients with COVID-19 hospitalised in Dr Soetomo General Hospital and Indrapura Field Hospital (Surabaya, Indonesia) for the TMPRSS2 p.Val160Met polymorphism. Polymorphism was detected using a TaqMan assay. We then analysed the association between the presence of the genetic variant and disease severity and viral load. We did not observe any correlation between the presence of TMPRSS2 genetic variant and the severity of the disease. However, we identified a significant association between the p.Val160Met polymorphism and the SARS-CoV-2 viral load, as estimated by the Ct value of the diagnostic nucleic acid amplification test. Furthermore, we observed a trend of association between the presence of the C allele and the mortality rate in patients with severe COVID-19.

**Conclusion:**

Our data indicate a possible association between TMPRSS2 p.Val160Met polymorphism and SARS-CoV-2 infectivity and the outcome of COVID-19.

**Supplementary Information:**

The online version contains supplementary material available at 10.1186/s40246-021-00330-7.

## Background

Coronavirus disease 2019 (COVID-19) is the biggest pandemic in the twenty-first century so far. Since the declaration of the pandemic by the World Health Organization (WHO), more than 110 million cases with more than 2.4 million deaths worldwide have been recorded as per mid-February 2021 [1]. COVID-19 is caused by an infection with the SARS-CoV-2 virus, which typically infects cells in the respiratory tract. The clinical presentations of COVID-19 range widely from asymptomatic infection to lethal pneumonia. It is known that three major factors, i.e. age, gender and the presence of underlying diseases, play a major role in determining COVID-19 severity [2–4]. However, it is not clear whether genetic variability contributes significantly to the clinical outcomes of COVID-19 patients.

One important factor that may play a crucial role in determining COVID-19 severity is the interaction between the virus and the host cells. SARS-CoV-2 infects the host cells by binding with its receptor on the surface of the host cell membrane. The main receptor for the SARS-coronavirus family is the angiotensin-converting enzyme 2 (ACE2) [5]. It is known that the spike (S) protein of SARS-CoV-2 mediates the binding of the virus to the ACE2 protein [6]. Considering the importance of virus receptor binding during the infection, it is logical to hypothesise that genetic variations within the gene encoding ACE2 may be associated with the degree of infection and hence the severity of the disease. Surprisingly, studies have reported no correlation between the genetic variations in the human *ACE2* gene and the severity of COVID-19 [7], as well as the previous severe acute respiratory syndrome (SARS) [8].

In addition to ACE2**,** several other molecules are also involved in the process of SARS-CoV-2 virus entry. For example, transmembrane protease serine 2 (TMPRSS2) [9] and neuropilin-1 (NRP1) [10] have been identified as co-receptors for SARS-CoV-2 that play a crucial role during virus entry. These molecules are important in mediating virus entry; for example, TMPRSS2 is known to facilitate the cleavage of the S protein, enabling membrane fusion and endocytic entry of the virus particles [11]. This has prompted us to hypothesi**s**e that genetic variability within the *TMPRSS2* gene may play a role in determining SARS-CoV-2 infection.

**A r**ecent analysis based on computational modelling suggested that out of more than eleven thousand single nucleotide polymorphisms (SNPs) within the human *TMPRSS2* gene (dbSNP, NCBI) only 21 SNPs with minor allele frequency (MAF) between 0.01 and 0.95 were predicted to affect the function of the protein [12]. Of these 21 SNPs**,** only two SNPs are missense variants (rs12329760 and rs75603675). The rs12329760 polymorphism (also known as p.Val160Met variant) has been shown in several studies to play a role in mediating the risk for prostate cancer, confirming clinical consequences of this genetic variant [13–16]**.** Therefore**,** in this study**,** we focused on studying the association between the p.Val160Met variant of the TMPRSS2 gene and the severity, viral load and clinical outcomes of COVID-19 patients. Although we did not find any correlation between the p.Val160Met polymorphism and disease severity, we observed a possible association between the TMPRSS2 pVal160Met variant and the viral load in COVID-19 patients.

## Results

### Characteristics of patients

Characteristics of COVID-19 patients included in this study are described in Table 1. Age distributions were significantly different between patients with asymptomatic and mild versus moderate and severe COVID-19. There was a significant difference in the sex distribution with a higher proportion of male patients in the symptomatic groups. Significant differences were observed in the proportions of patients with underlying diseases between the asymptomatic and mild patients versus moderate and severe groups. As expected, the patients in the asymptomatic and mild COVID-19 groups displayed a significantly lower frequency of underlying diseases compared with the moderate and severe groups, including diabetes (*P* value < 0.001), cardiovascular disease (*P* value = 0.009) and liver disease (*P* value = 0.007).

**Table 1:** Demographic and baseline characteristics

Variables	Asymptomatic (*N*=21)	Mild (*N*=12)	Moderate (*N*=32)	Severe (*N*=30)	All patients (*N*=95)	*P* value chi-square test (unless otherwise stated)
Age (years)	33.9 ± 2.4^*^	35.6 ± 2.7^*^	52.3 ± 2.1	48.8 ± 1.5	44.7 ± 1.3	^*^P<0.001 versus moderate and severe groups (ANOVA)
Gender (%)						
Male	8 (38.1%)	10 (83.3%)	19 (59.4%)	23 (76.7%)	60 (63.2%)	0.016
Female	13 (61.9%)	2 (16.7%)	13 (40.6%)	7 (23.3%)	35 (36.8%)	
Underlying diseases (%)						
Diabetes	0	0	9 (28.1%)	12 (40%)	21 (22.1%)	<0.001
CVD	2 (9.5%)	0	13 (40.6%)	10 (33.3%)	25 (26.3%)	0.009
Liver diseases	0	0	4 (12.5%)	9 (30%)	13 (13.7%)	0.007
Kidney diseases	0	0	4 (12.5%)	1 (3.3%)	5 (5.3%)	0.144
Lung diseases	0	0	3 (9.4%)	0	3 (3.2%)	0.107
Others	0	0	3 (9.4%)	2 (6.7%)	5 (5.3%)	0.386

### TMPRSS2 p.Val160Met polymorphism and COVID-19 severity

The TMPRSS2 p.Val160Met polymorphism (rs12329760) was successfully detected in all patients. The genotype and allele frequencies of this SNP are shown in Additional file 1. We observed a deviation of the allele frequency from Hardy–Weinberg equilibrium (*χ*^2^ = 6.72, *P* value = 0.035). However, the frequency of C allele (61.6%) and T allele (38.4%) in this study population seemed to be comparable with the reported frequency in the Asian population (dbSNP, NCBI) (Additional file 1).

The cross-tab analysis for the genotype and severity groups indicated no significant difference in the distribution of TMPRSS2 p.Val160Met polymorphism among the four groups of patients (Table 2). There were higher odds ratios in subjects with CC and CT genotypes to get symptomatic or more severe COVID-19 than those with TT genotype although they did not reach statistical significance (Table 3).

**Table 2:** Genotype and allele frequencies of the TMPRSS2 p.Val160Met polymorphism according to COVID-19 severity

	Asymptomatic (*N*=21)		Mild (*N*=12)		Moderate (*N*=32)		Severe (*N*=30)		Chi-square test
	*N*	%	*N*	%	*N*	%	*N*	%	
Genotype (amino acids)									
CC (Val/Val)	8	38.1	4	33.3	17	53.1	13	43.4	*χ*^2^= 3.11
CT (Val/Met)	7	33.3	5	41.7	11	34.4	10	33.3	*P*=0.79
TT (Met/Met)	6	28.6	3	25	4	12.5	7	23.3	
Allele									
C Allele	23	54.8	13	54.2	45	70.3	36	60	*χ*^2^= 3.51
T Allele	19	45.2	11	45.8	19	29.7	24	40	*P*=0.32

**Table 3:** Analysis of odds ratio for the risk of symptomatic or more severe COVID-19 in each genotype

	Asymptomatic (*N*=21) vs all symptomatic cases (*N*=74)			Asymptomatic and mild (*N*=33) vs moderate and severe cases (*N*=62)		
Genotype	Odds ratio	95% CI	*P*-value	Odds ratio	95% CI	*P*-value
CC	1.821	0.533–6.219	0.335	2.045	0.676–6.185	0.255
CT	1.592	0.447–5.664	0.471	1.432	0.462–4.437	0.573
TT	1	Reference		1	Reference	

### TMPRSS2 p.Val160Met polymorphism and viral load

**Fig. 1:**
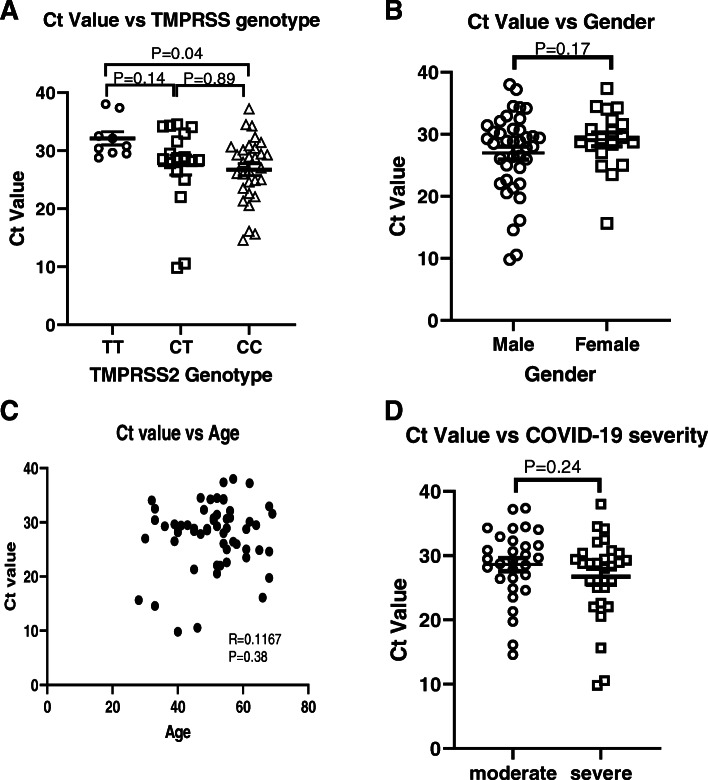
Analysis of NAAT Ct values of patients with moderate and severe symptoms. **a** Patients with CC genotype displayed significantly lower Ct values of NAAT compared with patients with TT genotype (ANOVA with post hoc multiple comparison test). **b** No difference was observed in Ct values between male and female patients. **c** There was no association between age and Ct value. **d** There was no difference in Ct value between patients with moderate and severe COVID-19

Next, we analysed the association between polymorphism and the viral load. All of the patients had positive results of the nucleic acid amplification testing (NAAT) for the SARS-CoV-2 virus. The Ct value was used as the semi-quantitative predictor of the viral load. Since Ct values vary depending on the qPCR system and the methodology of the NAAT, we only focused our analysis on patients with moderate and severe COVID-19. All of the patients in these groups were hospitalised in Dr Soetomo General Academic Hospital; hence, the NAAT was conducted in the same place, i.e. the Clinical Pathology and Microbiology Laboratory, Dr Soetomo General Academic Hospital. We analysed the Ct values of the first NAAT, which were conducted at the time when the patients were admitted to the hospital. A low Ct value is likely associated with a high viral load, whereas a high Ct value is likely to be associated with a low viral load. As illustrated in Fig. 1a, a significant difference was observed in the Ct value between patients with a TT genotype and patients with a CC genotype (*P* = 0.04), indicating a possible association of this genotype with a higher viral load. The Pearson correlation analysis also indicated a trend of decreasing Ct value with the presence of the C allele (*P* = 0.08). In contrast, we did not observe any difference and correlation between Ct value and patients’ gender (Fig. 1b) as well as between Ct value and age (Fig. 1c). Additionally, there was no difference in Ct value between patients with moderate and severe COVID-19 (Fig. 1d).

### TMPRSS2 polymorphism and patients’ outcome

During the course of the study, all of the patients with mild COVID-19 recovered, whereas 9.4% of the patients with moderate COVID-19 and 60% of the patients with severe COVID-19 died. When we analysed the association between TMPRSS2 p.Val160Met polymorphism and the patients’ outcomes, we did not find any association between the polymorphism and mortality in the moderate COVID-19 group (Table 4). However, we observed a trend of association in the severe group, in which a higher proportion of patients who died of COVID-19 had a CC genotype (*P* = 0.042 using the linear-by-linear association chi-squared test) (Table 4). We also observed an increasing trend of odds ratios of mortality in subjects with CC and CT genotypes (Table 5).

**Table 4:** Association between TMPRSS2 polymorphism with mortality/survival in patients with moderate-severe COVID-19

	Moderate COVID-19 (*N*=32)		Severe COVID-19 (*N*=30)	
	Recovered (*N*=29, 90.6%)	Died (*N*=3, 9.4%)	Recovered (*N*=12, 30%)	Died (*N*=18, 60%)
Genotype (amino acids)				
CC (Val/Val)	16 (50%)	1 (3.1%)	3 (10 %)	10 (33.3%)
CT (Val/Met)	10 (31.3%)	1 (3.1%)	4 (13.3%)	6 (20 %)
TT (Met/Met)	3 (9.4%)	1 (3.1%)	5 (16.7%)	2 (6.7%)
	Chi-square test *P*=0.498		Chi-square test *P*=0.109	
	Linear-by-linear chi-square association test *P*=0.299		Linear-by-linear chi-square association test *P*=0.042

**Table 5:** Analysis of odds ratio for the mortality in moderate and severe COVID-19 patients

	Odds ratio of mortality in moderate COVID-19			Odds ratio of mortality in severe COVID-19		
	Odds ratio	95% CI	*P*-value	Odds ratio	95% CI	*P*-value
Genotype						
CC	0.188	0.009-3.895	0.241	8.33	1.034-67.14	0.062
CT	0.3	0.014-6.382	0.423	3.75	0.473-29.75	0.335
TT	1	Reference		1	Reference	

## Discussion

This is the first study to demonstrate a possible association between TMPRSS2 p.Val160Met polymorphism and the degree of SARS-CoV-2 viral load as indicated by the Ct value of NAAT in patients with COVID-19. Patients with a CC genotype, which corresponds to the presence of valine amino acid, tend to display a lower Ct value (high viral load). We also found a trend of association between a CC genotype and mortality in a group of patients with severe COVID-19.

It is widely known that the SARS-CoV-2 virus enters the host cells via binding with ACE2, which acts as the main receptor for the viral particles [6, 9, 17]. The spike (S) protein of the SARS-CoV-2 virus consists of two sub-units: the S1 sub-unit, which is important for virus attachment, and the S2 sub-unit, which is essential for membrane fusion. ACE2 molecule can bind to the S1 protein to promote virus invasion into the host cells [6, 18]. In human, ACE2 is expressed in many organs, such as the upper respiratory tract, alveolar epithelial cells, vascular endothelial cells and macrophages [5].

In addition to ACE2, several other molecules are involved in SARS-CoV-2 virus binding and cell penetration. The S protein needs to be cleaved to activate the endocytic route of virus entry and to enable membrane fusion. It has been reported that several host proteases are involved in the process of S protein breakdown. These include TMPRSS2, cathepsin L, furin [9] and NRP1 [10].

TMPRSS2 is a serine protease that can prime the S protein of SARS-CoV-2 to enable cell penetration [9, 19]. The expression of TMPRSS2 in VeroE6 cells facilitates SARS-CoV-2 virus entry and promotes virus invasion [9]. Notably, treatment with the TMPRSS2 inhibitor (camostat mesylate) significantly reduced SARS-CoV-2 virus infection [9]. Moreover, TMPRSS2 is also involved in SARS-CoV-1 virus infection [20], supporting the idea of the critical role of this molecule in mediating virus entry.

The human *TMPRSS2* gene is located in chromosome 21.q22.3. It encodes protein that contains a transmembrane domain, low-density lipoprotein receptor class A (LDLRA) domain, scavenger receptor cysteine-rich (SRCR) domain and serine protease catalytic domain [21]. At least six nucleotide variants within the human *TMPRSS2* coding region that cause amino acid substitutions have been identified. These include p.Val160Met, p.Gly181Arg, p.Arg240Cys, p.Gly259Ser, p.Pro335Leu and p.Gly432Ala [22]. Of these variants, the p.Val160Met variant is often associated with diseases, notably prostate cancer. A study conducted on a Japanese population indicated that the TMPRSS2 p.Val160Met variant (also known as Met160Val polymorphism) was associated with the risk of sporadic prostate cancer [16]. Also, a study conducted on 214 patients with prostate cancer demonstrated that the T allele of this variant, which is associated with the presence of Met amino acid, was associated with TMPRSS2-ERG fusion and, thus, might be important in prostate cancer pathogenesis [23].

Our data indicate that in our study population the proportion of the genotypes deviates from the Hardy–Weinberg equilibrium. This deviation could be due to natural selection, non-random mating, genetic drift, or gene flow [24]. In our study population, the deviation was likely due to the higher number of subjects with CC and TT genotypes than the expected values (Additional file 1). However, the frequency of subjects with heterozygous genotype (CT) was lower than the expected value. Therefore, it is unlikely that the deviation was due to natural selection or advantages of a specific allele because the number of subjects with homozygous genotypes of both the T and C alleles was higher than the expected values, implying that there was no specific advantage of either the T or C allele. Thus, it is more likely that the deviation was due to non-random mating or genetic drift, which is more likely to occur in a small study population.

Recent bioinformatic analysis studying the functional effects of nucleotide variants within the human *TMPRSS2* gene revealed that the p.Val160Met variant was the most likely variant that might affect TMPRSS2 protein function and stability [12]. Furthermore, a computational analysis to predict the effects of polymorphism on protein structure suggested that the Val160Met substitution might create a pocket protein by influencing several amino acid residues, which might affect TMPRSS2 structure and its role in SARS-CoV-2 cell entry [12]. Possible changes in TMPRSS2 function and/or structure due to the Val160Met substitution might explain our findings on the association of this SNP with the viral load in COVID-19 patients. Alteration of TMPRSS2 function/structure will likely affect the binding of the S protein to ACE2 or the membrane fusion process. Reduction in TMPRSS2 enzymatic activity may decrease the furin cleavage of the S1 protein, which may subsequently decrease S2 fusion to the host’s cell membrane. However, further studies at the molecular level are required to prove this hypothesis, for example, by generating recombinant TMPRSS2 proteins bearing the variants and testing them in an in vitro model of SARS-CoV-2 cell infection.

Despite the association between pVal160Met polymorphism and the Ct value, we did not find any correlation between the variant and COVID-19 severity. This might be due to other confounding factors that strongly contribute to the severity of COVID-19. It is believed that factors, such as age [3], gender [25] and pre-existing diseases (hypertension, diabetes, CVD and lung disease) [4], strongly correlate with the risk of severe COVID-19. Further analysis with a larger study population is required to control these confounding variables. Interestingly, in patients with severe COVID-19, we observed a trend of association between this polymorphism and the mortality of COVID-19 patients. However, this requires further confirmation in studies with a larger sample size.

Several studies have found associations between genetic variations in the patient’s genome and COVID-19 severity. Many of the reported polymorphisms were related to genes involved in the development of inflammatory response, for example, polymorphisms in genes related to type 1 interferon immunity [26], polymorphisms in X-chromosomal TLR7 [27] and polymorphisms within genes involved in the interleukin 1 signalling pathway [28]. Our finding indicates a correlation between polymorphism in the gene encoding the virus receptor complex, i.e. TMPRSS2, and COVID-19 severity. Our data are consistent with previous reports on TMPRSS2 polymorphisms and COVID-19 severity. Initial whole genome analysis of 322 COVID-19 patients in a Chinese population observed a decreasing allele frequency of the TMPRSS2 rs12329760 variant among patients with severe disease compared with patients with mild COVID-19 [28], indicating the importance of TMPRSS2 in COVID-19. Consistently, studies on Italian COVID-19 patients also demonstrated the role of genetic polymorphisms within the *TMPRSS2* gene in determining COVID-19 severity. An observation on 133 COVID-19 patients found a difference in frequency of this variant in the COVID-19 patient cohort compared with the frequency in the reference databases [29]. In particular, in-depth analysis of available data from the COVID-19 Host Genetic Initiative (HGI) [30] suggested a significant association between TMPRSS2 gene polymorphism rs12329760 with severe/hospitalised COVID-19 [31]. However, further analysis on the available HGI data (https://app.covid19hg.org/) indicated that there was no significant association/difference of polymorphic allele between all SARS-CoV-2-infected subjects and the general population (*P*=0.569). This data is in line with our finding that the polymorphism may have more significant effects in severe cases of COVID-19.

Together, all of the data indicate a crucial involvement of the *TMPRSS2* genetic variation, the p.Val160Met (rs12329760) in particular, in mediating the severity of COVID-19. This will contribute to the growing body of evidence on the crucial involvement of the host’s genetic factor in determining susceptibility to and/or severity of COVID-19.

## Conclusions

In summary, this is the first study to demonstrate a possible association between TMPRSS2 p.Val160Met polymorphism and higher viral load in COVID-19 patients. The main limitation of our study is its small sample size. Further large-scale studies are required to validate our findings. Also, by using the Ct value, we can only have an estimate of the viral load. Precise determination of the viral RNA copy number using standard curve qPCR is required to accurately determine the viral load. Mechanistic analysis using a cell culture system is also important to confirm the effects of p.Val160Met on TMPRSS2 protein function. Nevertheless, our finding may provide new insights into the possibility of using this polymorphism as a biomarker or predictor for COVID-19 severity/clinical outcome. Furthermore, our data may also support the idea of targeting TMPRSS2 in COVID-19 therapy, as has been done in some clinical trials [32].

## Methods

### Study design, patients and data collection

This study was a cross-sectional study conducted from June to August 2020. During this period, a total of 95 patients with COVID-19 were enrolled. Patients with moderate and severe COVID-19 (*n* = 62, 65.3%) were hospitalised in Dr Soetomo General Academic Hospital, Surabaya, Indonesia, whilst 33 patients (34.7%) with asymptomatic or mild symptoms were treated in Indrapura KOGABWILHAN II Hospital, Surabaya, Indonesia. The diagnosis was confirmed using the nucleic acid amplification test (NAAT) of the oro-nasopharyngeal swab specimens. For patients with moderate and severe symptoms, the NAAT was performed in the Clinical Pathology and Microbiology Laboratory, Dr Soetomo General Academic Hospital, whereas for asymptomatic patients and patients with mild symptoms, the NAAT was conducted in the Centre for Health Laboratory, Surabaya, as part of the standard procedure for COVID-19 management in East Java Province, Indonesia. This study obtained ethical approval from the Local Ethics Committee of Dr Soetomo General Academic Hospital, Surabaya, Indonesia (0006/LOE/301.4.2/V/2020). All patients have signed the informed consent and agreed to participate in this study.

We clustered patients in three categories of disease severity based on criteria according to the WHO Guideline for COVID-19 Management [33] as follows: (i) mild: characterised by the presence of COVID-19 symptoms that meet the case definition of COVID-19 (fever, persistent cough, fatigue, anorexia, shortness of breath, myalgia, sore throat, nose congestion, headache, diarrhoea, nausea and vomiting, anosmia, ageusia) without evidence of viral pneumonia and hypoxia; (ii) moderate: characterized by the presence of the clinical signs of pneumonia but without any signs of hypoxia (SpO_2_ > 93%); and (iii) severe: characterized by the presence of the clinical signs of pneumonia and one of the clinical signs of respiratory distress (respiratory rate > 30×/min, severe respiratory distress, or SpO_2_ < 93%).

### DNA isolation

Heparinized peripheral blood samples were collected and stored in a −80^o^C freezer before use. DNA extraction was performed using the QIAamp® Blood DNA Midi kit (cat #51185, Qiagen) according to the manufacturer’s recommended protocol. DNA concentrations were determined using a microvolume spectrophotometer (NanoDrop Lite, Thermo Fisher Scientific). The procedures were conducted in the Biosafety Level 3 (BSL 3) Laboratory in the Institute of Tropical Disease, Universitas Airlangga, to reduce the risk of COVID-19 transmission.

### Polymorphism detection

The TMPRSS2 polymorphism (rs12329760, TMPRSS2 p.Val160Met also known as TMPRSS2 Met160Val polymorphism) was detected using a TaqMan SNP genotyping assay (Cat #4351379, Applied Biosystems, USA) in accordance with the protocol recommended by the manufacturer. Genotyping was performed using real-time polymerase chain reaction (RT-PCR) with VIC and FAM fluorescent reporters to indicate allelic discrimination. The 7500 Fast Real-Time PCR System (Applied Biosystems) was used in conjunction with the 7500 software v2.3 (Life Technologies™, Applied Biosystems) to create the allelic discrimination plot.

### Data analysis

Statistical analyses were performed using the IBM SPSS Statistics Software ver. 23 (IBM Corp.) or GraphPad Prism ver. 8 (GraphPad Software, LLC). A chi-squared test was used to examine the Hardy–Weinberg equilibriums and to determine the association between categorical variables in the cross-tabulation data. ANOVA with post hoc multiple comparisons was used to analyse numerical data. A *P* value less than 0.05 was considered to be statistically significant.

## Supplementary Information

**Additional file 1. **Genotype and allele frequencies of the TMPRSS2 p.Val160Met polymorphism in all patients**.**

## Data Availability

All data generated or analysed during this study are included in this published article and its supplementary information files.

## Abbreviations

COVID-19: Coronavirus disease 2019
SARS: Severe acute respiratory syndrome
SARS-CoV-2: SARS coronavirus 2
TMPRSS2: Transmembrane protease serine 2
ACE2: Angiotensin-converting enzyme 2
NRP1: Neuropilin-1
dbSNP: Database of single nucleotide polymorphism
NAAT: Nucleic acid amplification test
SNP: Single nucleotide polymorphism
LDLRA: Lipoprotein receptor class A
SRCR: Scavenger receptor cysteine-rich
CVD: Cardiovascular disease
HGI: Host genetics initiative
