# Supplementary material for: Initial study on TMPRSS2 p.Val160Met genetic variant in COVID-19 patients
Source: Hum Genomics. 2021 May 17;15:29. doi: 10.1186/s40246-021-00330-7 (PMC8127183; doi:10.1186/s40246-021-00330-7)
Supplement: Supplementary file 1 — Additional file 1. Genotype and allele frequencies of the TMPRSS2 p.Val160Met polymorphism in all patients. [file 40246_2021_330_MOESM1_ESM.docx]

**Supplementary table**

**Supplementary Table 1. Genotype and allele frequencies of the TMPRSS2 p.Val160Met polymorphism in all patients**

|  | Total patients (N=95) | | Hardy-Weinberg equilibrium |
| --- | --- | --- | --- |
|  | N (%) | Expected frequency |  |
| Genotype (amino acids) |  |  |  |
| CC (Val/Val) | 42 (44.2) | 36.02 | χ^2^= 6.72 |
| CT (Val/Met) | 33 (34.7) | 44.95 | *P* value = 0.035 |
| TT (Met/Met) | 20 (21.7) | 14.02 |  |
|  |  |  |  |
| Allele | N (%) | Reported frequency in Asian, East Asian and other Asian population (%)* | |
| C allele | 117 (61.6) | 60.9 |  |
| T allele | 73 (38.4) | 39.1 |  |

*Based on data on dbSNP (<https://www.ncbi.nlm.nih.gov/snp/rs12329760#frequency_tab>)
